# GeoTranMesh: a geometry-guided multi-branch mesh transformer for 3d liver segmentation

**DOI:** 10.1007/s11548-026-03600-8

**Published:** 2026-04-06

**Authors:** Jiaming Feng, Xukun Zhang, Shahid Farid, Sharib Ali

**Affiliations:** 1 AI in Medicine and Surgery Group, School of Computer Science, University of Leeds, LS2 9JT Leeds, UK; 2 Department of Diagnostic Radiology, Li Ka Shing Faculty of Medicine, The University of Hong Kong, Pok Fu Lam, Hong Kong; 3 Department of HPB and Transplant Surgery, St. James’s University Hospital, Leeds, UK

**Keywords:** Liver surface, 3D landmark segmentation, Mesh transformer, AR-guided surgery

## Abstract

**Purpose:**

Anatomical landmarks on the liver surface, such as the falciform ligament and hepatic ridge, exhibit complex geometry and significant morphological variability. Accurate segmentation of these structures on 3D liver mesh is essential for intraoperative navigation. This paper presents GeoTranMesh, a geometry-guided multi-branch mesh Transformer that employs hierarchical encoding–decoding and global geometric modeling to achieve high-precision liver mesh segmentation.

**Methods:**

A hybrid attention mechanism is proposed to fuse local geometric features with cross-branch contextual information, while a directional multi-branch fusion module refines features along tangent, normal, and bitangent directions to enhance geometric consistency. In addition, geometry-guided multi-task supervision, including boundary, distance, and normal regression, is incorporated to strengthen morphological feature learning.

**Results:**

On liver mesh dataset, GeoTranMesh achieved the highest segmentation accuracy, with Dice scores of 30.9% and 66.4% for the falciform ligament and liver ridge, respectively, an overall Dice of 59.5%, and a Chamfer distance of only 4.4 mm, demonstrating superior geometric consistency and anatomical precision.

**Conclusion:**

GeoTranMesh integrates hybrid attention and directional multi-branch fusion to enhance geometric consistency and morphological feature learning, achieving precise segmentation of complex anatomical landmarks, demonstrating potential for clinical and AR-guided liver surgery applications.

## Introduction

Augmented reality (AR)-based navigation can address several clinical challenges in laparoscopic or robotic liver surgery by facilitating overlay view of intricate liver anatomies such as blood vessels and tumor from preoperative 3D CT or MRI scans. However, such multi-modal fusion is challenging as they rely on anatomical regions such as liver’s ridge and the falciform ligament as registration constraints [1] in 2D laparoscopic images and 3D liver model extracted from CT/MRI scans. While several works [2–5] attempted segmentation of anatomical landmarks in 2D laparoscopic images to an acceptable accuracy, there are only a few recent studies [6–8] that attempted to perform segmentation in 3D liver mesh but with extremely low precision. This is an important step as it addresses the cumbersome and error-prone process of manual segmentation by surgeons in 3D model to establish fusion [3, 9]. One main bottleneck for achieving an accurate fusion is the variability in geometric topology of liver that inherently causes anatomical landmark inconsistencies. Fast, accurate, and automated landmark segmentation on 3D liver mesh can facilitate AR in surgery.

Previous fusion approaches used manual segmentation on 3D liver model and were mostly done by experts at their home institution on a few patients [3]. The P2ILF challenge [8] first introduced publicly available dataset of 11 patients incorporating 2D laparoscopic and 3D liver mesh anatomical segmentation paving the way toward data-driven approaches in 3D as well. The study reported large inaccuracies in 3D segmentation methods from four participating teams suggesting more research in this area. These teams explored point-based methods like PointNet2Plus [10] and mesh-specific strategies such as Mesh-CNN [11] for 3D landmark segmentation. Abbas et al. [7] proposed midline-constrained loss within PointNet2Plus architecture demonstrating geometric-based metric penalization can refine outliers improving mesh anatomy segmentation accuracy. Albeit, this approach improved overall Chamfer distance metric compared to the P2ILF teams, it still resulted in higher values with an average Chamfer distance of 16.58 mm. Recently, a Mesh-CNN approach was combined with graph-CNN at lower resolution first which was then mapped onto the high-resolution mesh surface in Zhang et al. [6]. This approach demonstrated improved accuracy on 3D liver segmentation compared to other previous approaches on Dice similarity metric. However, the Chamfer distance metric which provides more meaningful representation of thin 3D landmarks on liver mesh was higher (nearly 15 mm for ligament, and 5.5 mm for ridge), adversely affecting the 3D-2D registration.

In this paper, to address the challenges of accurately segmenting complex and morphologically variable liver surface landmarks in 3D liver model, we propose a geometry-guided multi-branch mesh Transformer (GeoTranMesh) for end-to-end segmentation on liver surface meshes. We would like to emphasize that our current work focuses on preoperative 3D liver surface landmark segmentation (ridges and falciform ligament) that are required to be accurate for successful preoperative to intraoperative registration to realize augmented reality in liver surgery [6]. To our knowledge, this is the first transformer-based method for 3D liver model landmark segmentation. Specifically, we introduce a GeoTranMesh framework: (1) a hierarchical encoder–decoder architecture that jointly models global shape semantics and local geometric details at the edge level; (2) a tri-branch refinement module that further enhances morphological feature learning through parallel *dilation*, *erosion*, and *boundary refinement* branches fed into a cross-branch interaction; (3) a directional attention fusion mechanism that adaptively integrates tangent, normal, and bitangent cues under geometry-aware constraints; and finally (4) multi-task prediction heads are used for prediction of seven different features that are utilized as regularization in our loss function. Extensive experiments on a manually annotated liver mesh dataset demonstrate that GeoTranMesh achieves superior accuracy and geometric consistency, outperforming representative mesh learning methods both quantitatively and qualitatively.

## Methods

**Fig. 1:**
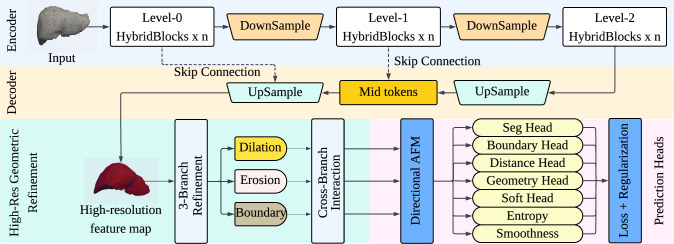
Overall framework of GeoTranMesh. The model adopts a hierarchical encoder–decoder that encodes mesh edges into multi-resolution tokens via *HybridBlocks* and token-based pooling. A tri-branch refinement module (*dilation*, *erosion*, *boundary*) performs high-resolution geometric modeling, and the Directional AFM fuses directional cues to produce the final features for multi-task prediction

We propose a geometry-guided multi-branch mesh Transformer (GeoTranMesh) for the segmentation of anatomical structures on the liver 3D mesh. As shown in Fig. 1, the model operates at the mesh edge level, taking geometric attributes as input and jointly predicting semantic classes and multiple auxiliary regression targets, including boundary, distance, geometric vectors, and soft labels. GeoTranMesh integrates a hierarchical geometry-aware encoder–decoder with a high-resolution multi-branch refinement module, capturing global contextual information while preserving fine boundary details.

For an input mesh $\mathcal {M} = (V, E)$, GeoTranMesh operates at the edge level. Each edge $e_i$ extracts its midpoint, tangent, normal, bitangent, curvature, and Laplacian spectral features to form a 52-dimensional feature vector $\textbf{x}_i \in \mathbb {R}^{52}$, whose composition is summarized in Table 1.

**Table 1:** Composition of the 52-D edge feature vector $\textbf{x}_i$

Group	Dim.	Description
*Base features (8-D)*		
Dihedral angle	1	Dihedral angle between the two incident faces
Symmetric opposite angles (sorted)	2	Two angles opposite to the edge in adjacent triangles
Symmetric height-to-base ratios (sorted)	2	Two ratios of triangle height to edge length
Edge midpoint	3	(*x*, *y*, *z*) coordinates of the edge midpoint
*Extended features (44-D)*		
Tangent	3	Normalized edge direction vector
Normal	3	Average normal of the two incident faces
Bitangent	3	$\textrm{cross}(\textrm{normal},\textrm{tangent})$
Edge length	1	Normalized edge length
Adjacent face areas	2	Areas of the two incident triangles
Neighborhood edge-length statistics	4	Mean / Std / Max / Min of neighboring edge lengths
Neighborhood angular cues	4	Cosine similarity between the edge tangent and up to four neighbor cues
Laplacian spectral features	8	First 8 Laplacian eigenvector features (precomputed and cached)
Heat Kernel Signature	16	Multi-scale HKS features (precomputed and cached)
**Total**	**52**

- **Hierarchical encoder–decoder.** A multi-layer *HybridBlock* encodes and decodes the mesh in a resolution-nested manner, producing high-resolution features with global contextual awareness.
- **High-resolution geometric refinement.** At the finest resolution, a tri-branch morphological module (*dilation*, *erosion*, *boundary*) refines features; cross-branch attention enables information exchange among branches.
- **Directional attention fusion (AFM) and multi-task heads.** Directional cues are fused to obtain the final representation, followed by heads for segmentation and auxiliary supervision; the network is trained end-to-end with a composite loss that combines classification, regression, and attention regularization.

### Mesh representation and neighborhood construction

GeoTranMesh builds its computational graph at the edge level, treating each edge as a node whose connections define local neighborhoods. Compared with vertex-based schemes, this design captures local surface orientation and boundary geometry more precisely. For each edge $e_i$, local geometric and spectral descriptors—midpoint, tangent, normal, bitangent, length, curvature, and spectral embeddings—are concatenated into a 52-dimensional feature vector $\textbf{x}_i \in \mathbb {R}^{52}$, preserving directional and local shape information. Each edge connects to neighboring edges sharing a vertex or face, forming an undirected graph $\mathcal {G} = (E, \mathcal {A})$, where the adjacency matrix $\mathcal {A}$ indicates whether two edges are connected (1 if adjacent, 0 otherwise). This graph encodes both topological continuity and geometric directionality, supporting consistent edge-level feature aggregation and attention.

### HybridBlock

**Fig. 2:**
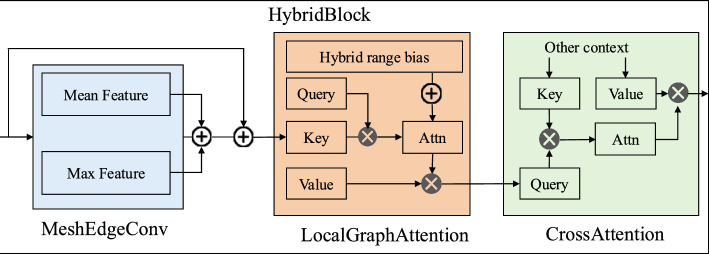
Architecture of the HybridBlock. The module consists of *MeshEdgeConv* (mean/max neighborhood aggregation), followed by *Local Graph Attention* with geometric bias, optional *Cross-Attention*, and a feed-forward network with residual normalization. Together, these components jointly model local geometric structures and directional correlations

The HybridBlock is the core encoding unit that models geometric and attentional features within local mesh neighborhoods (Fig. 2). Each block contains *MeshEdgeConv*, *LocalGraphAttention*, and optional *CrossAttention*, followed by residual normalization.

**MeshEdgeConv.** To model the local geometric distribution within each edge neighborhood, the features of adjacent edges $e_j \in \mathcal {N}(i)$ are aggregated using mean and max pooling, concatenated with the central feature, and linearly normalized to obtain the local convolutional feature:

1 $$\begin{aligned} \textbf{h}_i = \textrm{LN}\big (W_c [\,\textbf{x}_i,\, \textbf{f}_i^{\text {mean}},\, \textbf{f}_i^{\text {max}}\,]\big ). \end{aligned}$$

This operation performs adaptive convolution within the edge neighborhood, capturing topological continuity and local morphological variation.

**LocalGraphAttention.** Building upon the convolutional features, the HybridBlock models edge dependencies through multi-head attention. Each edge generates query, key, and value vectors $Q_i, K_j, V_j$, where the attention scores incorporate a geometric bias term $\phi (\cdot )$ based on relative position and tangent projection. The output feature is obtained by neighborhood-weighted aggregation:

2 $$\begin{aligned} \textbf{z}_i = \sum _{j \in \mathcal {N}(i)} \alpha _{ij} V_j. \end{aligned}$$

**CrossAttention (optional).** In multi-resolution or branch interaction stages, the HybridBlock employs CrossAttention, using the current feature as the query and cross-level or cross-branch features as keys and values to achieve cross-scale semantic alignment. The output is then updated through a feed-forward network with residual normalization, fusing geometry-aware convolution and directional attention:

3 $$\begin{aligned} \textbf{x}'_i = \textrm{LN}\big (\textbf{h}_i + \textbf{z}_i + \textrm{FFN}(\textbf{z}_i)\big ). \end{aligned}$$

### Hierarchical encoder–decoder with token-based pooling

GeoTranMesh employs a three-level hierarchical encoder–decoder structure to jointly model global shape and local geometric features across multiple scales. As illustrated in Fig. 1, the model starts from high-resolution edge-level features, performs two downsampling stages to obtain sparse tokens, and then progressively restores high-resolution representations through symmetric upsampling.

**Encoder.** The input features are linearly projected and normalized, then fed into Level-0, where stacked *HybridBlocks* extract local geometric features. Each level employs a *DownSampleBlock* that performs K-means clustering on edge midpoints to aggregate features into sparse tokens. Within each cluster, mean, max, and attention-weighted pooling are combined to generate the aggregated feature $\textbf{z}_t$. The resulting tokens are passed to the next level, progressively compressed to Level-2 to form a compact global geometric representation.

**Decoder.** Starting from Level-2, the decoder progressively upsamples features through *UpSampleBlocks*, mapping low-resolution tokens back to high-resolution representations. Each edge retrieves its coarse feature based on the cluster index $a_i$, which is concatenated with the corresponding encoder skip feature and linearly fused with normalization:

4 $$\begin{aligned} \textbf{x}^{(\textrm{up})}_i = \textrm{LN}\big (W_u [\,\tilde{\textbf{x}}_i,\, \textbf{x}^{(\textrm{skip})}_i\,]\big ). \end{aligned}$$

After two upsampling stages, high-resolution features $\textbf{h}_{\textrm{hr}}$ are obtained and fed into the subsequent *Tri-Branch Refinement* module. This hierarchical structure achieves a unified multi-scale representation of global and local geometric information.

### Tri-branch refinement with cross-branch interaction

**Fig. 3:**
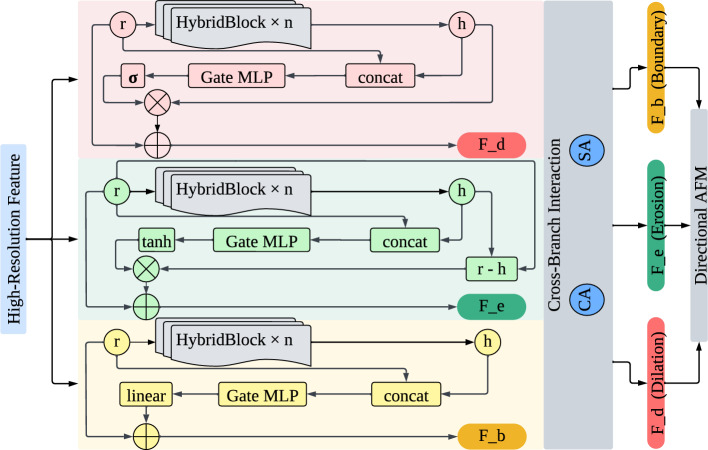
Illustration of the high-resolution geometric refinement stage

On top of the decoded high-resolution features $\textbf{h}_{\textrm{hr}}$, a tri-branch geometric refinement module is introduced to model three types of morphological responses—*dilation*, *erosion*, and *boundary*—as illustrated in Fig. 3. The three branches share the same neighborhood and geometric bias configuration. Each branch consists of several lightweight *HybridBlocks* and applies gated residual updates to refine its representation. Let the residual input be $\textbf{r}$, the intra-branch feature be $\textbf{u}$, and $G(\cdot )$ denote a gating function. The unified morphological update can be formulated as:

5 $$\begin{aligned} \textbf{h} = \textbf{r} + g(\textbf{r}, \textbf{u}), \end{aligned}$$

where

6 $$\begin{aligned} g(\textbf{r}, \textbf{u}) = {\left\{ \begin{array}{ll} \sigma (G([\textbf{r}, \textbf{u}]))\,\textbf{u}, &  \text {dilation}, \\ \tanh (G([\textbf{r}, \textbf{u}]))\,(\textbf{r} - \textbf{u}), &  \text {erosion}, \\ G([\textbf{r}, \textbf{u}]), &  \text {boundary}. \end{array}\right. } \end{aligned}$$

This yields three refined feature sets $\textbf{h}^{(d)}$, $\textbf{h}^{(e)}$, and $\textbf{h}^{(b)}$.

To enhance the consistency and complementarity among these morphological responses, *Cross-Branch Interaction* (CBI) is applied. For each branch $i \in \{d, e, b\}$, local self-attention is first performed within its neighborhood to produce $\tilde{\textbf{h}}^{(i)}$, followed by a crossattention step that integrates contextual information from the other branches through residual fusion:

7 $$\begin{aligned} \hat{\textbf{h}}^{(i)} = \textrm{LN}\!\left( \textbf{h}^{(i)} + \textrm{SelfAttn}(\textbf{h}^{(i)}) + \sum _{j \ne i} \textrm{CrossAttn}\!\big (\textbf{h}^{(i)} \leftarrow \textbf{h}^{(j)}\big ) \right) . \end{aligned}$$

All attention computations are modulated by the local neighborhood masks and geometric biases (consistent with the *HybridBlock*), thereby enforcing directional consistency during inter-branch alignment. The resulting representations $\{\hat{\textbf{h}}^{(d)}, \hat{\textbf{h}}^{(e)}, \hat{\textbf{h}}^{(b)}\}$ are then forwarded to the subsequent direction-aware fusion module.

### Directional AFM fusion with multi-task objectives

**Fig. 4:**
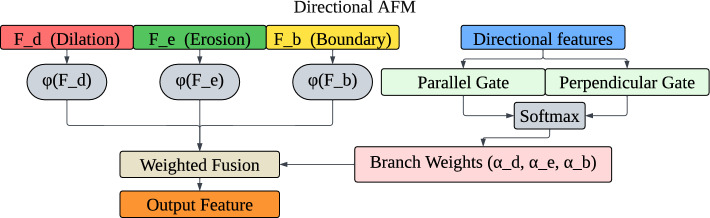
Directional attention fusion module (AFM)

To fuse the three-branch features under directional constraints, the Directional Attention Fusion Module (AFM) is introduced (see Fig. 4). For each edge *i*, a directional descriptor $\textbf{d}_i = [\,\textbf{t}_i,\, \textbf{n}_i,\, \textbf{b}_i\,]$ is constructed, where $\textbf{t}_i$, $\textbf{n}_i$, and $\textbf{b}_i$ denote the tangent, normal, and bitangent vectors, respectively. Two gating vectors, parallel gate $\textbf{g}^{\parallel }_i$ and the orthogonal gate $\textbf{g}^{\perp }_i$, adaptively modulate the fusion weights of the three branches. Given the branch features $\{\textbf{F}^{(d)}_i, \textbf{F}^{(e)}_i, \textbf{F}^{(b)}_i\}$, the fused representation is computed as:

8 $$\begin{aligned} \textbf{F}^{\text {fused}}_i = W_o\!\Big (\sum _{k \in \{d,e,b\}} \alpha _{i,k}\, \phi _k(\textbf{F}^{(k)}_i)\Big ) + W_r\, \textbf{F}^{\text {base}}_i. \end{aligned}$$

This module ensures directional consistency while achieving complementary integration across branches.

### Multi-task prediction heads

Based on the fused feature $\textbf{F}^{\text {fused}}$, five parallel heads are defined for segmentation, boundary, distance, geometry, and soft-label prediction. Training adopts class-weighted cross-entropy and Dice hybrid losses, with weighted regression for thin-structure supervision.

**Loss Function and Regularization.** Let $\boldsymbol{\alpha }_i$ denote the AFM branch weights. The total objective combines multi-task losses and directional regularization:

9 $$\begin{aligned} \begin{aligned} L&= w_{ce} L_{\text {CE}} + w_{dice} L_{\text {Dice}} + w_b L_{\text {BCE}} + w_d L_{\text {dist}} \\&\quad + w_g L_{\text {geo}} + w_s L_{\text {soft}} + w_t L_{\text {Tversky}} \\&\quad + \lambda _{ent}\, H(\boldsymbol{\alpha }) + \lambda _{sm}\, S(\boldsymbol{\alpha }), \end{aligned} \end{aligned}$$

where the entropy and smoothness regularization terms are defined as: $H(\boldsymbol{\alpha }) = -\frac{1}{N}\sum _{i=1}^{N}\sum _k \alpha _{i,k} \log (\alpha _{i,k} + \varepsilon ), \quad S(\boldsymbol{\alpha }) = \frac{1}{N}\sum _{i=1}^{N} \frac{1}{|\mathcal {N}(i)|} \sum _{j \in \mathcal {N}(i)} \Vert \boldsymbol{\alpha }_i - \boldsymbol{\alpha }_j\Vert _1.$ Here, $L_{\text {CE}}$ denotes the focal cross-entropy with label smoothing, $L_{\text {Dice}}$ is class-weighted Dice loss, $L_{\text {BCE}}$ is binary cross-entropy for boundary supervision (optionally up-weighted for target classes), $L_{\text {dist}}$ and $L_{\text {geo}}$ are Smooth-$L_1$ regressions, $L_{\text {soft}}$ is the KL-divergence distillation loss, and $L_{\text {Tversky}}$ is an optional class-specific Tversky term. This design fuses information under directional and branch-consistency constraints, enabling stable prediction of thin structures and fine boundaries through multi-task supervision. Hyperparameters $\lambda$ and *w* are experimentally set.

## Experiments and results

### Dataset

We evaluate GeoTranMesh on the same manually annotated liver surface mesh dataset as in Zhang et al. [6]. The internal cohort contains 200 liver surface meshes collected from three public abdominal CT datasets: 3Dircadb, LiTS, and AMOS. Liver surface models are extracted from the corresponding ground-truth liver segmentations using Marching Cubes in 3D Slicer, and further cleaned and simplified in MeshLab to ensure mesh quality (e.g., watertightness and manifoldness). As a result, mesh resolutions vary substantially across cases, ranging from approximately 3,000 to 20,000 edges. Two clinically relevant liver surface landmarks are annotated on each mesh: the falciform ligament and the ridge. Annotations are provided as vertex-level regions on the mesh surface using Blender and are reviewed by clinical experts.

For evaluation, we split the 200 meshes into training/validation/testing sets with a 10:3:7 ratio (100/30/70). In addition, to assess cross-dataset generalization, we further test on an external cohort from the MICCAI’2022 P2ILF challenge [8] (9 cases).

### Competing methods and experimental setup

To validate the effectiveness of GeoTranMesh, we compare it with several representative point- and mesh-based learning methods, including PointNet++ [10], DGCNN [12], MeshCNN [11] and its enhanced variant MeshCNN-1 (with additional edge-midpoint coordinates), TSGCN [13], as well as the previous two-stage model Nested Resolution Mesh-Graph CNN (MeshGraphCNN) [6].

All methods are implemented in PyTorch and trained under the same hardware configuration (NVIDIA L40S GPU). We use the AdamW optimizer with an initial learning rate of $1\times 10^{-3}$ and a cosine annealing learning rate schedule. The batch size is set to 1 and all models are trained for 150 epochs. Model selection follows an early-stopping strategy, and the checkpoint with the best validation Dice score is used for final evaluation. Unless otherwise specified, all results are reported using the same evaluation metrics and experimental protocol.

### Computational efficiency

Our method targets *preoperative* liver surface landmark annotations and thus does not require real-time intraoperative inference. Nevertheless, efficiency is still important to reduce preoperative workload and enable interactive review. Table 2 summarizes model complexity and inference latency. We report the *pure network forward-pass* latency measured using CUDA events (10 warm-up iterations and 50 timed iterations, repeated twice) on the LiTS-80 mesh (5,634 edges). The peak GPU memory usage is 3.58 GB during training and 345 MB during inference with single-precision floating-point of 32, and batch size of 1.

**Table 2:** Model complexity and inference latency comparison

Method	#Params (M)	Latency (s/mesh)	GPU
PointNet++	1.735	0.107	RTX 8000
DGCNN	1.454	**0**.**007**	RTX 8000
MeshCNN	**0**.**982**	0.493	RTX 8000
MeshCNN-1	**0**.**982**	0.574	RTX 8000
TSGCN	4.128	0.083	RTX 8000
MeshGraphCNN	1.454	0.267	RTX 8000
GeoTranMesh (ours)	2.832	0.863	L40S

### Metrics for comparison

Model performance is evaluated using two standard metrics: the Dice Similarity Coefficient (Dice) and the 3D Chamfer Distance (CD, mm). Dice measures the overlap between predicted and ground-truth regions, while CD quantifies the geometric alignment between the predicted and annotated surfaces. All metrics are computed on the test set, where a higher Dice and lower CD indicate better segmentation accuracy and boundary quality.

### Comparison of results by different methods

Table 3 summarizes the quantitative comparison results for the falciform ligament (Ligament), liver ridge (Ridge), and overall performance. Overall, the ridge shows higher segmentation accuracy than the ligament, reflecting the greater difficulty of modeling the latter due to its thin structure and fuzzy boundaries.

*PointNet++* and *MeshCNN* failed to detect the ligament, while *MeshCNN-1* achieved moderate improvement by incorporating edge-midpoint geometry. *DGCNN* and *MeshGraphCNN* further enhanced performance through dynamic and multi-resolution geometric modeling, respectively. In comparison, the proposed *GeoTranMesh* achieved the best results in both Dice and CD metrics (Ligament 30.9%, Ridge 66.4%, Overall 59.5%, CD 4.4 mm). On CD, our approach is 36.23% lower than the second best method demonstrating the superiority of geometrical consistency and anatomical accuracy of our approach.

**Table 3:** Comparison of different segmentation methods on *Ligament*, *Ridge*, and overall performance

	Ligament		Ridge		Overall	
Method	Dice $\uparrow$ (%)	CD $\downarrow$ (mm)	Dice $\uparrow$ (%)	CD $\downarrow$ (mm)	Dice $\uparrow$ (%)	CD $\downarrow$ (mm)
PointNet++[10]	0.0±0.0	60.3±18.2	20.7±16.5	33.4±24.2	19.1±15.2	37.1±22.5
DGCNN[12]	21.2±23.5	22.1±18.7	57.2±12.8	5.6±6.0	50.3±15.1	8.0±6.8
MeshCNN[11]	0.0±0.1	165.1±55.6	0.6±**2**.**0**	85.0±51.4	0.8±2.3	86.1±49.8
MeshCNN-1[11]	16.2±13.8	33.6±19.5	49.7±16.9	12.2±12.1	42.5±14.2	15.0±9.2
TSGCN[13]	1.8±7.2	34.8±25.8	35.9±19.0	22.7±23.0	33.3±17.7	27.1±24.9
MeshGraphCNN[6]	**32**.**6**±17.9	14.9±12.3	59.6±13.8	5.5±4.8	54.9±13.5	6.9±4.8
**GeoTranMesh**	30.9±**17**.**0**	**9.1±5.5**	**66**.**4**±11.1	**3.3±2.2**	**59.5±10.5**	**4.4±2.3**

“Overall” represents the combined segmentation evaluation of the falciform ligament and the liver ridge

**Fig. 5:**
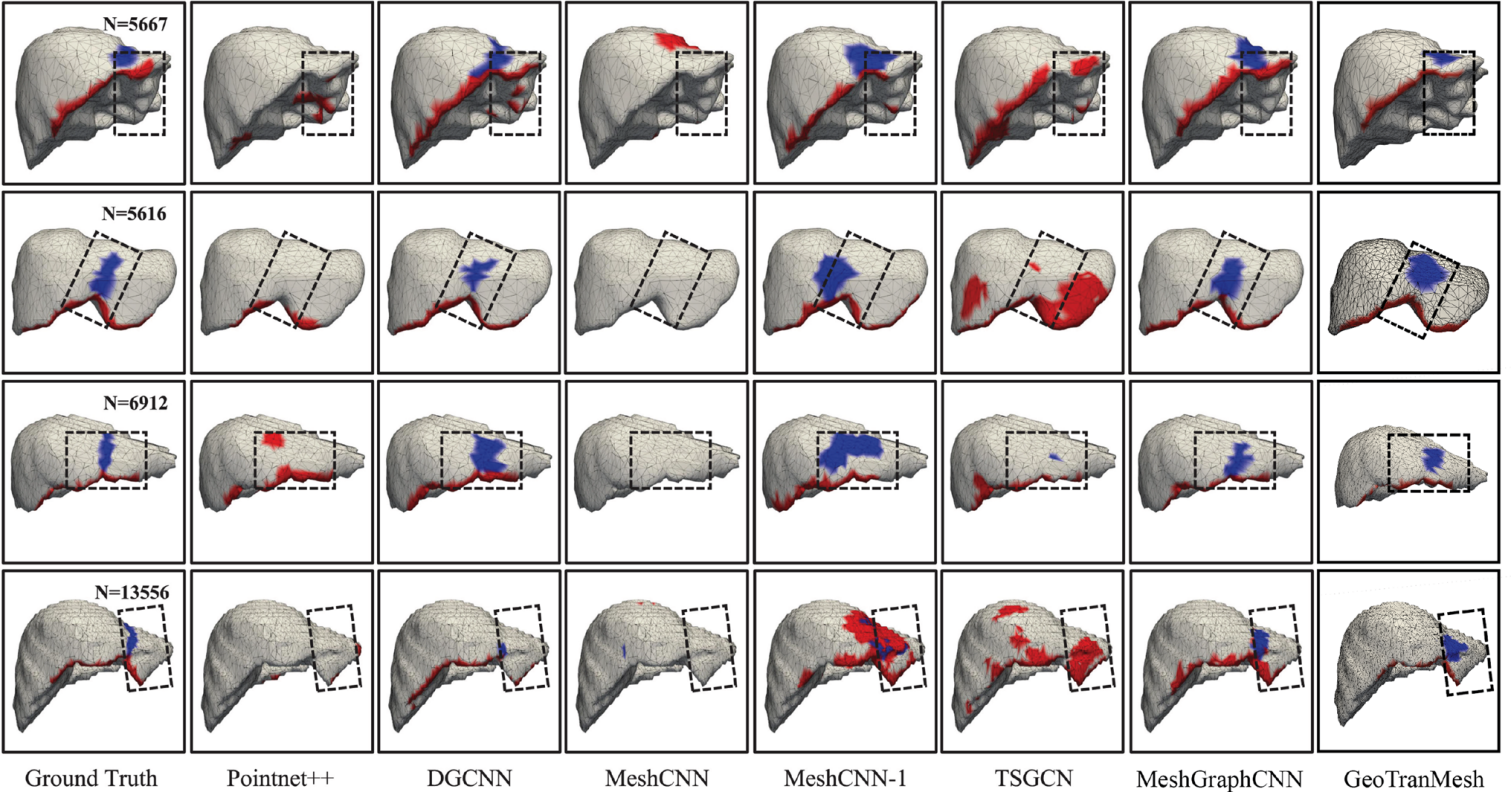
Comparison of segmentation results from different methods. The falciform ligament is shown in blue, the liver ridge in red, and dotted boxes highlight key regions

Figure 5 illustrates qualitative results across meshes of different resolutions. *PointNet++* and *MeshCNN* failed to identify the falciform ligament, while *MeshCNN-1* and *TSGCN* showed partial improvement but still suffered from over- and under-segmentation. *DGCNN* and *MeshGraphCNN* produced more stable overall results, though the predicted boundaries remained coarse. In contrast, *GeoTranMesh* achieved more stable segmentation across multiple resolutions, exhibiting more precise boundary reconstruction and consistent morphology, particularly for the liver ridge region. Visual validation by a clinical expert indicated that the results from the GeoTranMesh provided anatomically faithful, accurately localized annotations improving interpretability and its usefulness for reproducible preoperative planning and intraoperative orientation.

### Ablation study

To evaluate the contribution of each module, we report an ablation study in Table 4. Removing branch interaction or the directional adaptive fusion module (w/o Cross-Branch, w/o AFM) leads to performance degradation, indicating that inter-branch information interaction and weighted fusion are essential for effective feature integration. Using only the dilation or erosion branch (CBI + dilation only, CBI + erosion only) results in blurred boundaries or missing details, respectively, verifying the complementarity of the three branches in morphological refinement. Regarding the loss design, disabling the Smooth *L*1-loss (w/o Smooth *L*1-loss) weakens cross-scale feature propagation and geometric constraints, thereby affecting overall performance.

Removing the tri-branch refinement module (w/o Tri-Branch Refinement) significantly reduces segmentation accuracy and increases Chamfer Distance, with the Overall Dice dropping from 59.5 to 52.63% and the Overall CD increasing from 4.4 to 7.24 mm, demonstrating that this morphological refinement structure plays a key role in boundary consistency and geometric precision. Meanwhile, retaining only the main segmentation head and removing all multi-task prediction heads (w/o Multi-task Heads, Seg head only) also results in substantial performance degradation (Overall Dice 52.30% and Overall CD 7.75 mm), indicating that multi-task supervision can effectively enhance the modeling of fine-grained structures and geometric consistency. The full model achieves the best Dice and CD scores, confirming that the synergy of all modules jointly improves geometric precision and boundary consistency.

**Table 4:** Ablation study of key components of our full GeoTranMesh framework

	Ligament		Ridge		Overall	
Experiment	Dice$\uparrow$	CD $\downarrow$	Dice$\uparrow$	CD$\downarrow$	Dice$\uparrow$	CD$\downarrow$
	(%)	(mm)	(%)	(mm)	(%)	(mm)
w/o Cross-Branch	29.6	9.36	59.89	3.83	54.14	6.59
w/o AFM	29.0	10.26	59.39	3.72	53.62	6.99
CBI + dilation only	27.5	10.75	58.32	4.05	52.46	7.40
CBI + erosion only	31.0	9.41	61.30	**3**.**22**	55.54	6.32
w/o Smooth *L*1-loss	**31**.**5**	10.45	61.61	3.54	55.89	6.99
w/o Tri-Branch Refinement	27.5	10.36	58.63	4.11	52.63	7.24
w/o Multi-task Heads	26.9	11.53	58.57	3.97	52.30	7.75
**GeoTranMesh (ours)**	30.9	**9**.**1**	**66**.**4**	3.3	**59**.**5**	**4**.**4**

**Fig. 6:**
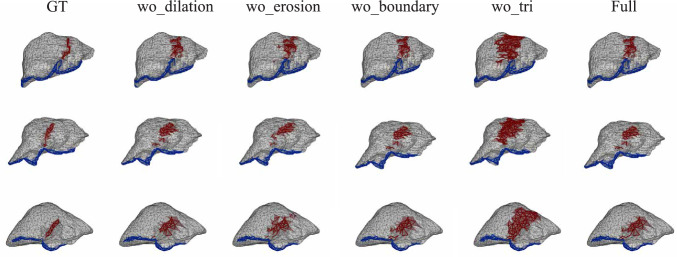
Qualitative ablation visualization of the tri-branch refinement module. Columns from left to right: GT, w/o dilation, w/o erosion, w/o boundary, w/o tri-branch, and full model (GeoTranMesh)

**Qualitative ablation visualization.** To further verify the distinct contributions of the three branches in the proposed tri-branch module, Fig. 6 visualizes representative cases comparing the ground truth (GT), variants that remove a single branch (w/o dilation, w/o erosion, w/o boundary), the variant without the entire tri-branch module (w/o tri), and the full model. Removing different branches leads to different error patterns. For example, disabling the dilation or erosion branches tends to aggravate under or over-segmentation respectively, while removing the boundary branch primarily degrades contour fidelity. Similarly, removing the entire tri-branch module yields the most noticeable degradation, whereas the full model produces the closest alignment to the GT.

### Generalizability assessment on external dataset

To assess the generalizability of GeoTranMesh, we evaluated the model on nine external liver mesh datasets from the MICCAI 2022 P2ILF challenge, with mesh resolutions ranging from 3k to 13k edges. Due to intraoperative visibility constraints, these meshes contain only partial annotations and exhibit notable geometric and topological variations compared with our training data. Given this incompleteness, we primarily report the 3D Chamfer Distance (CD, mm), which is the official metric of the P2ILF challenge. As summarized in Table 5, GeoTranMesh achieved CD values of 16.6 ± 1.5 mm for the falciform ligament, 15.9 ± 2.5 mm for the liver ridge, and 15.8 ± 2.3 mm overall, outperforming existing methods. These results demonstrate that GeoTranMesh maintains stable performance across varying mesh resolutions and partially visible surfaces, suggesting strong cross-dataset generalizability.

**Table 5:** External test results on P2ILF dataset (3D Chamfer Distance, mm)

Method	Ligament (mm)	Ridge (mm)	Overall (mm)
PointNet++[10]	–	60.5±41.8	65.7±43.2
DGCNN[12]	60.2±36.4	24.3±13.3	27.1±12.3
MeshCNN[11]	–	88.5±53.7	80.7±42.9
MeshCNN-1[11]	67.8±46.1	38.3±19.4	42.9±14.3
TSGCN[13]	45.8±0.0	70.5±46.7	74.5±46.3
MeshGraphCNN[6]	36.3±22.7	19.0±12.5	18.8±8.6
**GeoTranMesh**	**16.6±1.5**	**15.9±2.5**	**15.8±2.3**

“–” indicates missing ligament annotations making Chamfer Distance (CD) not computable

## Discussion and conclusion

This paper presents GeoTranMesh, a geometry-guided multi-branch mesh Transformer for the segmentation of anatomical landmarks on the liver surface. Experimental results demonstrate that the proposed method outperforms existing approaches in both Dice and Chamfer Distance metrics, particularly achieving higher geometric accuracy and morphological consistency for the hepatic ridge. The edge-level representation and hierarchical encoder–decoder framework enable multi-scale geometric modeling, while the tri-branch refinement and directional fusion effectively enhance boundary details and structural consistency, improving the modeling of complex surface geometry. However, performance remains limited for extremely thin and sparse classes such as the falciform ligament, which are particularly sensitive to class imbalance and morphological variability. Although our framework already incorporates class-weighted supervision, the remaining errors indicate that under-representation can still be a key bottleneck. In future work we will investigate more targeted solutions, including class-balanced re-weighted loss and sampling strategies for minority or hard examples, as well as midline- or centerline-constrained weighted loss formulations [7]. Overall, GeoTranMesh achieves notable improvements in geometric precision and boundary fidelity, demonstrating strong potential for clinical use and augmented reality–guided liver surgery applications.

## Data Availability

The source code is publicly available at https://github.com/aimsgroup-Leeds/GeoTranMesh.
